# Comprehensive review on the pathogenesis of hypertriglyceridaemia-associated acute pancreatitis

**DOI:** 10.1080/07853890.2023.2265939

**Published:** 2023-10-09

**Authors:** Minhao Qiu, Xiaoying Zhou, Maddalena Zippi, Hemant Goyal, Zarrin Basharat, Mateusz Jagielski, Wandong Hong

**Affiliations:** a Department of Gastroenterology and Hepatology, the First Affiliated Hospital of Wenzhou Medical University, Wenzhou, Zhejiang, People’s Republic of China; b Department of Gastroenterology and Hepatology, the First Affiliated Hospital of Wenzhou Medical University, Wenzhou, Zhejiang, People’s Republic of China; c Unit of Gastroenterology and Digestive Endoscopy, Sandro Pertini Hospital, Rome, Italy; d Department of Surgery, University of TX Health Sciences Center, Houston, TX, United States; e Independent Researcher, Islamabad, Pakistan; f Department of General, Gastroenterological and Oncological Surgery, Nicolaus Copernicus University in Toruń, Poland

**Keywords:** Hypertriglyceridemia, lipids, acute pancreatitis, pathogenesis, mechanism

## Abstract

It is well known, that the inflammatory process that characterizes acute pancreatitis (AP) can lead to both pancreatic damage and systemic inflammatory response syndrome (SIRS). During the last 20 years, there has been a growing incidence of episodes of acute pancreatitis associated with hypertriglyceridaemia (HTAP). This review provides an overview of triglyceride metabolism and the potential mechanisms that may contribute to developing or exacerbating HTAP. The article comprehensively discusses the various pathological roles of free fatty acid, inflammatory response mechanisms, the involvement of microcirculation, serum calcium overload, oxidative stress and the endoplasmic reticulum, genetic polymorphism, and gut microbiota, which are known to trigger or escalate this condition. Future perspectives on HTAP appear promising, with ongoing research focused on developing more specific and effective treatment strategies.

## What’s known in the literature

1. Reports of lipid metabolism disorders, in particular hypertriglyceridaemia and how this can be one of the causes of acute pancreatitis, are increasingly numerous in the literature.
2. The pathogenesis of this aforementioned association has not yet been fully and exhaustively described to date.

## What’s new in this article?

1. We give a brief overview of the triglyceride metabolism and animal models of hypertriglyceridaemia associated acute pancreatitis, which may be help for readers to understand the mechanisms and future research directions.
2. Furthermore, to try to provide an exhaustive explanation of this pathogenetic relationship, we analyze several factors that do not only concern the inflammatory process.

## 1. Introduction

Pancreatic acinar cells, with their related enzyme secretion, play a pivotal role in the onset of the inflammatory process that characterizes acute pancreatitis (AP), which can evolve into local pancreatic complication, systemic inflammatory response syndrome (SIRS) [1]. Analysis of estimates from the Global Burden of Disease Study 2019 revealed a 62.9% increase in the global incidence of AP and a 64.8% increase in mortality compared to 1990 [2]. AP is estimated to have an incidence of 34 affected individuals per 100,000 person/year, with an ever-increasing global trend [3].

Among the most common dyslipidemias, there is certainly hypertriglyceridaemia (HTG), which occurs when plasma triglyceride levels are greater than 1.7 mmol/L, i.e. 150 mg/dL [4–6]. The guideline on lipid treatment designated the classification of HTG: serum triglyceride levels 150 to 199 mg/dL, borderline high; 200 to 499 mg/dL, high; and very high or severe, having levels ≥500 mg/dL [7]. According to the guidelines of the American College of Gastroenterology (ACG), serum triglycerides can represent a favourable cause for the development of AP, only when their levels are higher than 1000 mg/dL (>11.3 mmol/L) [8]. The incidence of hypertriglyceridaemia-associated AP (HTAP) has increased considerably over the last twenty years [9–11]. Jin et al. have demonstrated that the HTAP incidence increased from 14.0% in 2001 to 34.0% in 2016 [12]. However, the exact prevalence of HTAP might be underestimated because serum TG was not measured in large numbers of patients with HTAP, and many newly discovered ones were not referred to specialized clinics [13]. HTAP is often recurrent with more frequent local and systemic complications leading to higher mortality rates than pancreatitis caused by other factors [13–17]. Song et al. identified that HTG, male gender, and diabetes were independent risk factors of recurrent AP [18]. However, the underlying pathogenesis of HTAP is still not fully deduced. It follows that the incomplete knowledge on this topic has limited both the research and the subsequent development of therapies aimed at the treatment of this pathology.

Animal models have proposed several theories on the pathogenesis of HTAP [19–23]. One of these, for example, postulates that the onset of pancreatitis is triggered by excessive exposure of pancreatic acinar cells to high levels of free fatty acids (FFA), exerting toxic consequences through various mechanistic processes [24]. Another theory states that elevated levels of some bulky lipoproteins lead to increased plasma viscosity [24–26]. This last, exacerbated by acidosis, leads to capillary occlusion and ischaemia, ultimately leading to pancreatitis [25,27,28].

**Figure 1.**
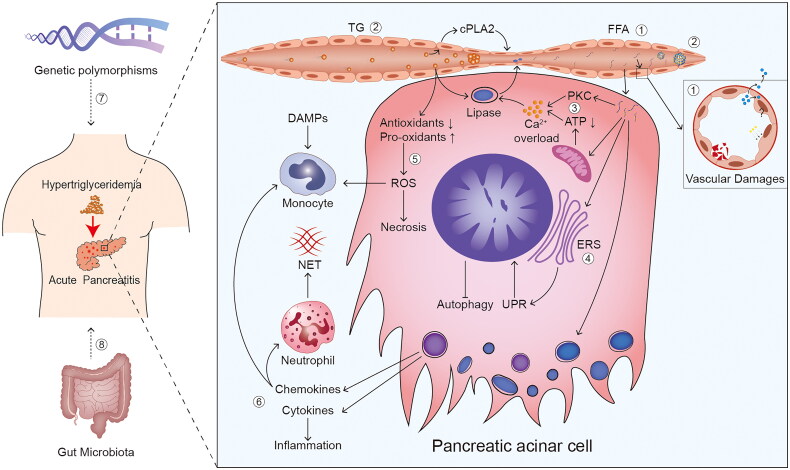
**Overview of potential mechanisms of hypertriglyceridaemia induced and aggravated AP.** ① free fatty acids (FFAs) and ② microcirculatory disorder are considered vital factors in the mechanisms of hypertriglyceridaemia (HTG) induced acute pancreatitis (AP). microcirculatory disorder is primarily characterised by injuries caused by vasoconstriction/vasospasm, deceleration of blood flow, and blockage of blood vessels. ③ calcium (Ca^2+^) overload, ④ endoplasmic reticulum stress (ERS), ⑤ oxidative stress, ⑥ chemokines and cytokines, ⑦ genetic polymorphisms, and ⑧ gut microbiota are considered the potential mechanisms of HTG aggravated AP. Reactive oxygen species (ROS) are the main acting components in oxidative stress. Abbreviation: TG: triglyceride; cPLA: cytoplasmic phospholipase A; PKC: protein kinase C; ATP: adenosine triphosphate; UPR: unfolded protein responses; ROS: reactive oxygen species; DAMPs: damage-associated molecular patterns; NET: neutrophil extracellular trap.

However, these two theories may not provide a comprehensive explanation of the process by which HTG induces and aggravates AP. In this article, we present a comprehensive review of current knowledge on HTAP pathogenesis, including the role of FFA, microcirculatory disorder, calcium (Ca^2+^) overload, endoplasmic reticulum stress, oxidative stress, chemokines and cytokines, genetic polymorphism, and gut microbiota **(**Figure 1**)**. Because of the difficulty to obtain human pancreatic tissue during an AP episode, animal models were often used to study the early cellular events during AP. In order to help readers easily understand the mechanisms and future research directions, we also give a brief overview of the triglyceride metabolism and animal models of HTAP.

## 2. Triglyceride metabolism

**Figure 2.**
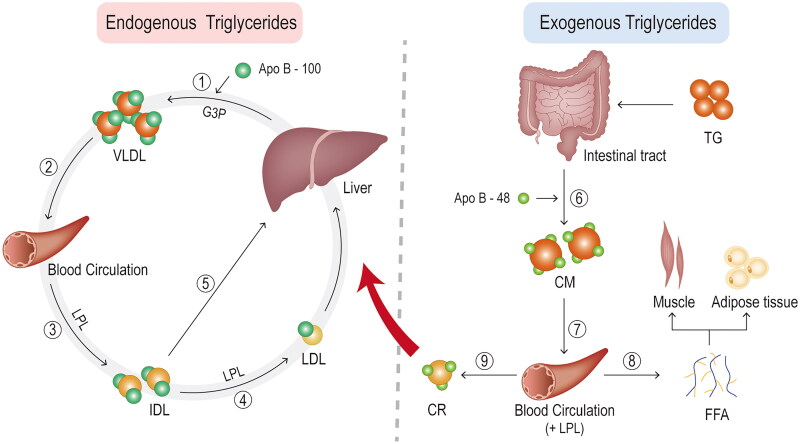
**Triglyceride metabolism.** ① endogenous triglycerides are synthesised from free fatty acids and glycerol in hepatocytes *via* the glycerol-3-phosphate pathway. Together with apolipoprotein (apo) B-100, they form VLDL particles. ② packaged triglyceride in the form of VLDL is then secreted into the blood circulation. ③ the VLDL particles are hydrolysed by lipoprotein lipase (LPL) in the plasma, producing progressively smaller particles and, eventually, intermediate density lipoprotein (IDL) particles. ④ some IDL particles undergo further catabolism in the blood by LPL or by hepatic lipase to generate low-density lipoprotein (LDL) particles. ⑤ others are taken up by hepatic cells and catabolised directly through binding to the LDL receptor or LDL receptor-related protein on hepatocytes. ⑥ exogenous dietary triglycerides are eventually absorbed by enterocytes (mainly in the small intestine) after a series of reactions in the intestinal tract, where they combine to apo B-48 to form chylomicrons (CM). ⑦ CM takes a rather circuitous route into the blood circulation. In the blood, CM is quickly hydrolysed by LPL along the luminal surface of the capillaries, resulting in the production of free fatty acids (FFA) and chylomicron remnants (CR). ⑧ the FFA enters the cells and is oxidised, not only for muscle energy but also for resynthesis with glycerol into triglycerides and stored in adipose tissue. ⑨ CR enters the hepatic circulation through a similar elimination pathway to some IDLs, by binding to LDL receptors or LDL receptor-related proteins.

Triglycerides play a key role in efficiently storing the body’s excess energy [29]. Triglyceride-rich lipoproteins contain both endogenous and exogenous triglycerides **(**Figure 2**)**. The very low-density lipoproteins (VLDL) and the chylomicrons (CMs) represent the two main types of triglyceride-rich lipoproteins, which are respectively secreted by the liver and by the intestine, serve for transport endogenous and exogenous (dietary) lipids to peripheral tissues. Both triglyceride-rich lipoproteins contain apolipoprotein (apo) B as their primary apolipoprotein. They also contain other apolipoproteins, such as apo A-V, apo C-II, apo C-III, and apo E, some of which derive from circulating high density lipoprotein (HDL) [5].

### 2.1. Triglyceride synthesis

The principal pathway for triglyceride synthesis is glycerol-3-phosphate occurring in the liver and adipose tissue. The last accounts for more than 90% of the total triglyceride synthesis [30]. Triglycerides are synthesized in hepatocytes from FFAs and glycerol, and subsequently, in synergy with apo B, VLDL particles are assembled .Finally, triglycerides are packaged into VLDL and secreted into the plasma. Through the secretion of the hepatocytes, the apo C-I, C-II, C-III and E are incorporated on the surface of VLDL particles [31].

The intestinal absorption by the enterocytes of triglycerides, cholesterol and long-chain saturated and unsaturated fatty acids occurs through the formation of micelles. On the other hand, short and medium chain fatty acids (chain length from 2 to 10 carbon atoms) are able to directly cross enterocyte membranes and, as a consequence, immediately reach the bloodstream. Thereafter, intestinal enterocytes synthesize triglycerides from the absorbed fatty acids and package them in CMs, i.e. lipoprotein particles capable of transporting fats and cholesterol from the small intestine throughout the body [32]. Before entering the bloodstream through the thoracic duct, CMs are transported by the peri-mesenteric lymphatic vessels and the superior vena cava. There is no certain explanation as to why these formations take such a tortuous path. Likewise, this route could be useful to protect the liver from a high lipid load and to allow a greater supply of fatty acids to organs such as the heart, lungs and various peripheral tissues [33]. During this crossing, CMs engulf apo C-II, C-III and E [31].

These particles, by binding to the lipoprotein lipase (LPL) expressed on the luminal surface, undergo a metabolization process which it serves to convert triglycerides into FFA, thus allowing their absorption by peripheral tissues. Consequently, this overabundance of lipids inevitably leads to an accumulation of triglycerides in the heart, skeletal muscle and liver. Furthermore, lipoproteins, rich in triglycerides, may represent a source of atherosclerotic lipids, and in addition, the same lipolysis of triglycerides could lead to the production of lipids toxic to the vessels [33].

### 2.2. Triglyceride breakdown

LPL is an essential enzyme in mediating the hydrolysis of triglyceride-rich lipoproteins, thereby enabling the release of FFA and residual lipoproteins. Lipase maturation factor 1 (LMF1) is required to synthesize the active enzyme, while angiopoietin-like protein 4 is able to inhibit its activity by cleaving the LPL dimer [33]. Once LPL reaches the surface of endothelial cells, it binds to glycosylphosphatidylinositol-anchored HDL-binding protein 1 and is secreted into the bloodstream, supplying the tissues with energy [35]. LMF1 is required for LPL secretion from adipocytes and myocytes and its’ absence causes severe HTG [36]. The activation of LPL requires the intervention of apo C-II, whose activity is regulated by various proteins, including apo C-III and apo A-V, as well as proteins 3 and 4 which are similar to the angiopoietin and which are well known factors of inhibition of LPL [5,34].

LPL hydrolyzes the VLDL particles in the plasma, progressively reducing their size until arriving at the formation of intermediate density lipoproteins (IDL). The catabolism of IDL occurs partly in the hepatocytes by LPL and hepatic triglyceride lipase to generate LDL particles, and partly in the blood. The action of the cholesterol ester transfer protein allows VLDL and LDL particles to acquire additional cholesterol esters in plasma, through the exchange of triglycerides in VLDL and LDL for cholesterol esters in HDL [31]. Along the luminal capillary surface, CMs are rapidly hydrolyzed by LPL resulting in the production of FFA and CM remnants [31].

Residual lipoproteins, absorbed by the vascular endothelium, are able to promote inflammation and atherosclerosis through various mechanisms, including abnormal secretion by endothelial cells and reduced flow-mediated dilatation [37]. The liver, using apo E as a ligand, takes up both CM residues through LDL receptors and related proteins [5]. A similar elimination occurs for the remnants of VLDL, although some undergo further hydrolysis by liver lipase to produce LDL, which is a substance composed entirely of cholesterol esters and apo B. What remains of unhydrolyzed VLDL, is absorbed by the liver and serves as a source of lipids for the subsequent synthesis of VLDL triglycerides. Other sources for the synthesis are FFAs, released both from adipose tissue under the action of hormone-sensitive lipase and from the de novo formation of hepatic lipogenesis stimulated by the consumption of simple sugars [5]. The released fatty acids can enter cells without the aid of receptors or protein transporters, such as fatty acid transport proteins or platelet glycoprotein 4 [33]. Different types of cells are capable of oxidizing FFAs, for example, skeletal and myocardial myocytes, furthermore these particular lipids are resynthesized through glycerol into triglycerides and finally stored in this form in the adipose tissue. The CM residues, rich in cholesterol esters and apo E, after binding to LDL receptors or LDL receptor-related proteins expressed on hepatocytes, are eliminated from the circulation [31].

## 3. Animal models of HTAP

Animal models are still the primary means of conducting research on the pathogenesis of diseases due to medical ethics requirements. Animal models have many advantages: Firstly, the crucial events in the pathophysiology of HTAP can be studied in animal models [38]. Secondly, the distinct, reproducible, and measurable process could provide a way of exploring the early stages of AP with hypertriglyceridaemia and subsequent progression [39]. HTG is one of the most common aetiologies of AP; however, high-quality studies are still lacking in explaining the HTAP mechanism, which can be attributed to the lack of appropriate animal models [40].

### 3.1. Selection of experimental animals

The most common experimental animals used for HTAP studies are rats [6,41–43], mice [40], hamsters [44], and LPL gene-deficient animals (e.g. LPL-deficient mice) [45,46]. Of these, Sprague-Dawley rats are the most commonly used experimental animal to study lipid metabolism, and many researchers have used Sprague-Dawley rats to build HTAP animal models [6,41–43]. Nevertheless, no study has been published using Wistar rats as a model for HTAP despite wide implementation in cholesterol metabolism studies. By comparing the serum and liver lipid responses of Sprague-Dawley rats and Wistar rats to a high-fat diet, Udomkasemsa et al. concluded that Sprague-Dawley is more suitable for building hypertriglyceridaemia associated AP models [47].

### 3.2. The method of establishing a model

The administration of high-fat diet is mainly used to induce HTG in animal models (Table 1) [6,19,40,48–51]. The intraperitoneal injection of poloxamer 407, a non-ionic surface active agent with lower physiological toxicity, has been reported to establish hypertriglyceridaemia in animal models [40]. Loginova et al. reported that poloxamer 407 causes a significant increase in serum cholesterol and triglyceride, with a higher effect on triglyceride than cholesterol [52]. Despite not mimicking the naturally occurring hyperlipemia state, this approach is currently widely utilized due to its advantages, including rapidity, simplicity, controllability, and usefulness [39]. Furthermore, HTG can also occur in transgenic animals due to genetic defects without unique feeding methods.

**Table 1.** Modelling of hypertriglyceridaemia associated AP animals.

Pan et al. 2017 [170]	Q. Zhang et al. 2019 [51]	Hong et al. 2020 [19]	Yang et al. 2020 [6]	Mei et al. 2020 [50]	Dai et al. 2021 [49]	J. Wang et al. 2022 [48]	Author and Year of Publication
C57BL/6 mice (male, 20-25 g): The HTG model was induced by intraperitoneal injection of P-407 (every other day, 0.5 g/kg), and the AP model was induced by intraperitoneal injections of caerulein (10 times at a 1-h interval, 50 μg/kg).	SD rats (male, 4 weeks): The HTG model was induced by fed with a high-fat diet (2% cholesterol + 15% lard + 0.2% sodium cholate + 82.8% normal fat diet) for 4 weeks, and the AP model was established *via* intraperitoneal caerulein injection (7 times at a 1-h interval, 50 μg/kg).	SD rats (male, 200-220 g): The HTG model was induced by fed with a high-fat diet for 8 weeks, and the AP model was subsequently induced by a standardized retrograde infusion of sodium taurocholate solution (5%) into the biliary-pancreatic duct.	SD rats (male, 8 weeks): The HTG model was induced by intraperitoneal injection of P-407 (every day, 0.25 g/kg) for 14 days, and the AP model was subsequently induced by intraperitoneal injections of L-arginine (twice at a 1-h interval, 2.5 g/kg).	SD rats (male, 120-140 g): The HTG model was induced by fed with a high-fat chow (3% cholesterol + 20% lard + 77% normal chow diet) for 2 weeks, and the AP model was subsequently induced by intraperitoneal injections of caerulein (4 times at a 1-h interval, 50 g/kg).	C57BL/6 mice (male, 6-10 weeks): The HTG model was induced by intraperitoneal injection of P-407 (every other day) for 4 weeks, and the AP model was subsequently induced by intraperitoneal injections of caerulein (10 times at a 1-h interval, 100 μg/kg). SD rats (male, 120-150 g): The HTG model was induced by fed with a high-fat diet (3% cholesterol + 20% saturated animal fat, lard + 77% normal chow), and the AP model was induced by intraperitoneal injections of cerulein (2 times at a 1-h interval, 50 μg/kg).	SD rats (4 weeks, male): The HTG model was fed with a high-fat diet (0.2% sodium cholate + 2% cholesterol + 15% saturated animal fat + 82.8% standard rat chow) for 4 weeks, and the AP model was subsequently induced by retrograde injection of sodium taurocholate into the biliary-pancreatic duct (4%, 1 mL/min/kg).	Animals and Methods
The serum levels of TG, and histopathological scores of the pancreas and pulmonary were higher in HTG mice than normal mice. The duration and extent of HTG was positively correlated with the severity of AP.	The mean serum amylase levels, TG levels, and pancreatic histology scores were higher in experimental groups.	The extent and severity of the pancreatic injury and the pancreatic damage scores were significantly higher in the experimental groups than in the control group. Pancreatic LPO and MDA activity, and serum FFA levels were much higher in the experimental groups, but pancreatic SOD activity and GSH content were lower.	Serum levels of TNF-α, IL-6, AMY, ALT, AST, BUN, and Cr were significantly higher in the experimental groups than the control groups. The pancreatic histological scores of experimental groups were markedly higher.	Serum TG concentration and serum TC concentration were significantly increased. The HFD AP group exhibited significantly increased plasma amylase and lipase levels and elevated serum inflammatory cytokines TNF-α, IL-1β, and IL-6. The injury of pancreatic tissues was severe. MPO levels were also increased significantly.	HTG reduces tissue repair capacity after AP and aggravates pancreatic inflammation.	Serum TG concentration and pancreatic histopathological scores were higher in the experimental group than the normal group.	Results

HTG: hypertriglyceridaemia; SD: Sprague-Dawley; AP: acute pancreatitis; TG: triglyceride; P-407: Poloxamer 407; TC: total cholesterol; FFA: free fatty acid; HFD: high-fat diet; MDA: malondialdehyde; GSH: reduced glutathione; SOD: superoxide dismutase; LPO: lipid peroxidation.

Although there are various methods for inducing AP, only three of them (caerulein injection, bile acid infusion, and L-arginine injection) have been confirmed in establishing the HTAP model [39]. It is known that subcutaneous or intraperitoneal caerulein injection is one of the most commonly used methods to induce AP in rodents [38] as it is simple, non-invasive, and applicable in multiple species. Moreover, these effects are also highly reproducible and stable. However, caerulein is expensive and induces only mild AP, which can limit its application in pancreatitis research [53].

The retrograde biliopancreatic duct infusion of bile acids can induce moderate to severe AP in animal models. It is based on the theory of severe AP caused by the reflux of biliary and pancreatic juices in the pancreatic duct. However, the pancreatitis induced by this method and is associated with high mortality making it cumbersome to use and limiting its application [39]. Lastly, intraperitoneal L-arginine injections can give rise to AP as well as can control the degree of pancreatic necrosis by adjusting its dose and duration and simulate multiple organ failure syndrome in AP [53]. However, there is a significant variation in individual response after AP establishment, with mice exhibiting less stability than rats and different mice strains exhibiting varying pancreatic injury [54].

## 4. Potential mechanisms of hypertriglyceridaemia induced and aggravated AP

### 4.1. Free fatty acid

Triglycerides are not inherently toxic to the pancreas. The pancreatic lipase breaks down triglycerides into FFAs causing lipotoxicity [55]. The severity of pancreatitis depends on the inflammatory response and the injury caused by lipotoxicity [56]. Large triglyceride-rich lipoproteins in HTG were associated with AP severity and progression [57].

It is known that FFAs are of two types based on the presence or absence of C-C double bonds: 1) saturated fatty acids (SFA) and 2) unsaturated fatty acids (UFA). Dietary unsaturated/saturated components determine the fatty acid composition in fat cell triglyceride, affecting AP severity [58]. Cellular excess long-chain SFA is considered to be associated with lipotoxicity [59]. The most prevalent SFA includes palmitic acid (PA) and stearic acid. PA is the most abundant FFA in the human body [60] and can promote protein kinase activation, Ca^2+^ overload, endoplasmic reticulum stress, increased production of reactive oxygen species (ROS), and recruitment of macrophages [21,61,62]. Ben-Dror and Birk showed that exposing pancreatic exocrine primary cells to normal or high levels of PA results in significantly elevated levels of pancreatic lipase protein and transcription [21].

On the other hand, among monounsaturated and polyunsaturated FFA, oleic acid and linoleic acid are the most prevalent in the body. Oleic acid has been found to be the most abundant FFA found in pancreatic necrotic collections [63]. UFA produce higher levels of FFA as they are more easily lipolysis by pancreatic lipase [57,58]. The better aqueous UFA stability helps them exist as monomers at higher concentrations than SFA, resulting in lipotoxic inflammation and organ failure [58,64]. Notably, UFA caused a sudden release of intracellular calcium, inhibition of mitochondrial complexes I and V, upregulation of inflammatory mediators and acinar necrosis causes acute pancreatitis [65]. However, protective effects of oleic acid for SFAs-induced lipotoxicity have been reported in beta cells, rat islets, and high fat diets-induced obese rats [66].

**Figure 3.**
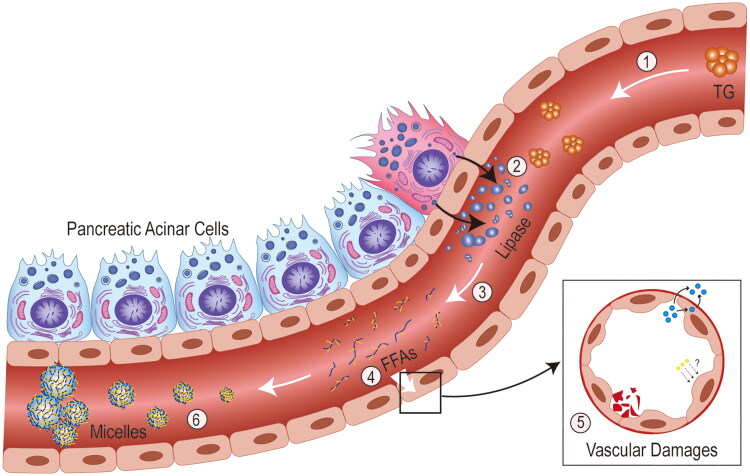
**Mechanism of action of free fatty acids.** ① excess triglycerides reach the vascular bed of the pancreas with blood transport in the form of triglyceride-rich lipoproteins. ② for idiopathic reasons, large amounts of lipase from pancreatic acinar cells are released into the blood through the vascular endothelium. ③ triglycerides are broken down by lipase into fatty acids and glycerol. ④ free fatty acids (FFAs) that exceed the albumin-binding capacity act directly on the vascular endothelium, ⑤ causing vascular damage such as endothelial dysregulation, vascular leakage and coagulation activation. ⑥ high concentrations of FFAs gradually aggregate into micelles with detergent like properties, causing ischaemia and subsequently triggering acidosis, trypsin activation, etc.

FFA can interact with extracellular Ca^2+^ causing hypocalcaemia in severe AP. This could be one of the possible mechanisms of HTAP progression as early Ca^2+^ supplementation delays organ failure in severe AP models [67]. Apart from the binding capacity of albumin, high levels of circulating FFA can also aggregate into micelles with detergent-like properties which can cause local ischaemia, trigger acidosis, activate lysosomal cathepsin-B and trypsinogen to form trypsin, leading to pancreatic autodigestion [39,68]. Moreover, FFAs produce a direct cytopathic effect on acinar and vascular endothelial cells, resulting in endothelial dysfunction and vascular leakage. It could also cause activation of the coagulation cascade by inflammatory pathways, endoplasmic reticulum stress, increased oxidative stress, and apoptosis [69,70] (Figure 3). Furthermore, FFAs directly engage with toll-like receptors (TLR) and induce nuclear factor kappa B (NF-κB)-dependent production of inflammatory cytokines such as tumour necrosis factor (TNF)-α and interleukin (IL)-6 [2371]. Therefore, FFAs are one of the key factors in developing HTAP (Table 2) [21,50,62,65,72–74].

**Table 2.** The roles of FFAs in pancreatic cells.

Navina et al. 2011 [65]	Chang et al. 2015 [74]	Danino et al. 2015 [73]	Ben-harosh et al. 2017 [72]	Nemecz et al. 2018 [62]	Ben-dror & birk, 2019 [21]	Mei et al. 2020 [50]	Author and year of publication
Pacs	Pacs	Exocrine pancreas ar42j and pacs	Pscs	Pancreatic beta cells	Exocrine pancreas ar42j and pacs	Pacs	Cell type
Ufa: lla, la, oa Sfa: sa, pa	Ufa: la, palmitoleic acid, dha, aa, oa Sfa: pa, sa	Ufa: oa Sfa: pa	Ufa: oa Sfa: pa	Ufa: oa Sfa: pa	Ufa: oa Sfa: pa	Pa	Fatty acids
The ufas generated from lipolysis contributed to necrosis, inflammation, multisystem organ failure, and mortality in ap. The rapidity of that ufa caused inhibition of mitochondrial complexes i and v, the release of intracellular calcium, inflammatory mediator up-regulation and acinar necrosis, contributing to injuries of renal and lung, and even mortality.	High concentrations (1 mmol/l) of ufas induced a rise in concentrations of cytosolic ca^2+^ in acinar cells and caused cell damage and intra-acinar cell trypsin activation. Conversely, low concentrations (0.1 mmol/l) of ufas, both concentrations of sfas and triglycerides did not induce a rise in cytosolic ca^2+^ concentrations.	Pa exposure induced an elevation in endoplasmic reticulum stress markers (e.g. Xbp1, chop and bip). Oa exposure did not induce any endoplasmic reticulum stress or elevation in levels of inflammation markers. They may be considered as being ‘protective’ during the processes.	High concentration (500 μm) of sfas at the endoplasmic reticulum membrane damaged transporters and membrane receptors, and promoted endoplasmic reticulum stress through upr sensors: atf4, chop, xbp1, and grp78, leading to disturbance in cell homeostasis. Tnf-α protein levels and il-6 transcript were found elevated in pscs treated with pa. Activation of upr, endoplasmic reticulum stress, and elevation in endoplasmic reticulum stress markers were not found in pscs treated with oa but only increased expression of il-6 and tnf-α.	Pa exposure induces oxidative stress by increasing ros production and inducing endoplasmic reticulum stress. This causes a significant increase in levels of il-8 and il-6. Oa attenuated apoptosis, endoplasmic reticulum stress, ros production, inflammation, and up-regulation of proinflammatory cytokines (il-8, il-6), upr transcription factors and chaperone bip.	Acute exposure to pa had a deleterious effect on the endoplasmic reticulum stress of pacs and resulted in a pro-inflammatory state. The state was expressed by elevation in the transcript of tgf-β, tnf-α, and il-6, as well as the protein levels; Exposure to monounsaturated oa had a protective effect on endoplasmic reticulum stress, expressed by a significant reduction in tnf-α transcript. Exposure to a combination of mono-ufa and sfa protects pacs from endoplasmic reticulum stress response induced by sfas.	The blocking-up of autophagy flux was found in acinar cells treated with pa.	Main finding

PACs: pancreatic acinar cells; PA: palmitic acid; UFA: unsaturated fatty acid; OA: oleic acid; SFA: saturated fatty acid; TNF-α: tumour necrosis factor-α; IL-6: interleukin-6; TGF-β: transforming growth factor-β; ROS: reactive oxygen species; FFA: free fatty acid; UPR: unfolded protein response; Bip: heavy-chain binding protein; IL-8: interleukin-8; PSCs: pancreatic stellate cells; CHOP: C/EBP homologous protein; Xbp1: X-box binding protein 1; GRP78: glucose regulated protein 78kD; ATF4: activating transcription factor 4; LA: linoleic acid; AA: arachidonic acid; DHA: docosahexenoic acid; Ca^2+^: calcium; LLA: linolenic acid; SA: stearic acid.

### 4.2. Microcirculatory disorder

**Figure 4.**
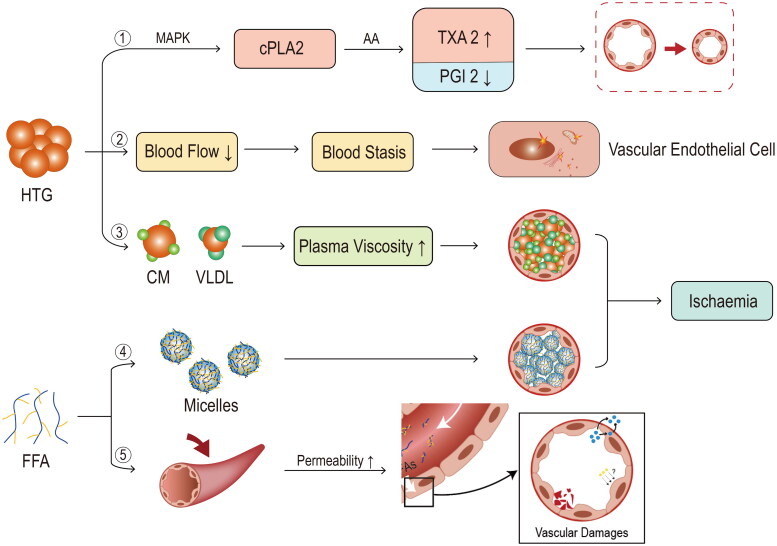
**Mechanisms of microcirculatory disorders.** ① following the onset of hypertriglyceridaemia (HTG), progressive activation of cytoplasmic phospholipase A (cPLA), release of arachidonic acid (AA) and thromboxane A2 (TXA2)/prostaglandin (PGI2) imbalance through activation of p38 mitogen-activated protein kinase (MAPK), ultimately leads to vasoconstriction/vasospasm. ② stasis may occur after blood flow is slowed by HTG velocity, which in turn affects the ultrastructure of the vascular endothelium. ③ with an increase in triglyceride-rich lipoproteins, plasma viscosity rises, which can eventually lead to blockage of blood vessels. ④ as mentioned previously, free fatty acids (FFA) accumulate to form micelles, which can also block blood vessels. ⑤ FFA also acts directly on the pancreatic vasculature, causing vascular damage through increased permeability.

The pathological changes of pancreatic microcirculatory disturbance in HTAP are complex, including local secretion of vasoactive factors, increased vascular permeability, ischaemia/reperfusion, intravascular coagulation, and leukocyte adherence [75] (Figure 4). Boulet et al. demonstrated that binding triglyceride-rich lipoproteins to platelet receptors can affect multiple agonist-dependent signalling pathways necessary for platelet activation and aggregation [76]. The p38 mitogen-activated protein kinase (MAPK) pathway activation is most critical, which activates cytosolic phospholipase A2 which is an enzyme catalyzing arachidonic acid (AA) release from membrane phospholipids [77]. Ultimately, the release of the vasoconstrictor thromboxane A2 (TXA2) and vasodilator prostaglandin (PGI2) would occur. The TXA2/PGI2 imbalance results in excessive capillary bed contraction and pancreatic microcirculation aggravation [78]. In addition, the secretion of thromboxane B2, a powerful vasoconstrictor induced by platelet-neutrophil complex, caused pancreatic ischaemia and worsened pancreatitis [79].

Plasma hyperviscosity is also involved in the pathogenesis of HTAP [24,25,27,80]. HTG can slow pancreatic blood flow affecting pancreatic vascular endothelial cells ultrastructure and microcirculation damage [81–83]. In particular, irregular and intermittent perfusion, even static blood flow, and abnormal projections were observed inside the narrowed vascular lumens in pancreatic animal models [83]. Theoretically, an increase in larger CMs increases plasma viscosity [84]. Increased blood viscosity reduces tissue microcirculation and can cause cerebral or cardiovascular ischaemia [85,86]. Elevated plasma viscosity can also result in the obstruction of pancreatic capillaries and subsequent ischaemia, which can lead to cellular acidosis. The increased acidosis may increase the risk of local thrombosis, embolism, and trypsinogen activation by cathepsin B, thereby exacerbating inflammation [25,27,87]. However, there was no direct evidence of pancreatitis caused by hyperviscosity in patients with hypergammaglobulinemia and polycythaemia, which are the most common causes of hyperviscosity. This indicates a low risk of hyperviscosity syndromes complicated by pancreatitis. Additionally, micelles with detergent properties formed by FFA can cause blockage of blood vessels, leading to vascular ischaemia, embolism, and reperfusion injury, resulting in an acidic environment that exacerbates toxicity and acidosis [70,78]. FFAs can have a direct damaging effect on vascular endothelial cells, causing intravascular coagulation and vascular endothelial disorders particularly context of pancreatitis [70].

### 4.3. Ca^2+^ overload

#### 4.3.1. Ca^2+^ overload and AP

**Figure 5.**
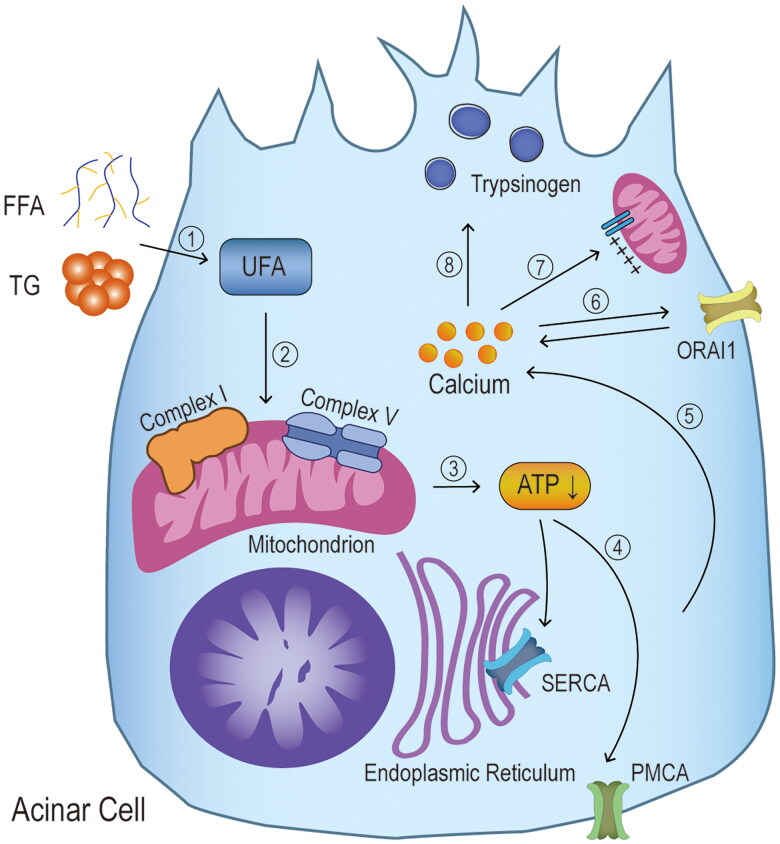
**Mechanisms of Ca^2+^ overload.** ① high triglycerides and FFAs act as an irritant and cause pathological increases in intracellular calcium ions. Among these are unsaturated fatty acids (UFA), ② which cause dysfunction of mitochondrial complexes I and V. ③ the mitochondrial impairment leads to a reduction in ATP production, ④ followed by a progressive inhibition of the two ATP-dependent calcium channels (sarcoendoplasmic reticulum Ca^2+^ ATPase (SERCA) pumps and plasma membrane Ca^2+^ ATPase (PMCA) pumps), ⑤ preventing the normal clearance of intracellular calcium ions and eventually increasing their concentration. ⑥ the increase in calcium concentration causes calcium release-activated calcium channel protein 1 (ORAI1) to promote calcium ion entry into the cell, maintaining high toxic levels of calcium ion concentration. ⑦ the overload of calcium ions causes the permeable transition pore of the mitochondria to open in a state of high electrical conductivity, leaving the membrane potential required for ATP production deficient. ⑧ persistently elevated calcium ions most importantly causes elevated trypsinogen levels, and subsequent premature activation.

Cytosolic Ca^2+^ influx is necessary for pathological and physiological responses in acinar cells (Figure 5). Excessive intracellular Ca^2+^ results in mitochondrial permeability transition pore opening which is present in the inner and outer mitochondrial membranes. When the pore is in a high conductance state, the membrane potential required for adenosine triphosphate (ATP) generation is lost, ultimately resulting in mitochondrial permeabilization. The loss of mitochondrial membrane permeabilization is known to be a universal trigger for cell death. Moreover, ATP depletion increases the toxic Ca^2+^ concentration by disrupting the ATP-dependent sarcoendoplasmic reticulum Ca^2+^ ATPase (SERCA) pumps and plasma membrane Ca^2+^ ATPase (PMCA) pumps from clearing excessive cytosolic Ca^2+^. It impairs cytoprotective mechanisms that need ATP, such as autophagy and unfolded protein responses (UPR) [88,89].

#### 4.3.2. Ca^2+^ overload and HTAP

Earlier studies have shown that an increase in intracellular Ca^2+^ concentration is significantly associated with lipid metabolism disorders and can be inhibited by pre-treatment with inhibitors of Ca^2+^ adenosine triphosphatases and chelating agents of intracellular Ca^2+^ depots [65,90,91]. Chang et al. first demonstrated that the high concentrations of UFA and SFA induce increased cytosolic Ca^2+^ concentrations in acinar cells. These Ca^2+^ concentrations correlated with a dose-dependent UFA/SFA ratio [74]. Acinar cells are injured only when high concentration of triglycerides is hydrolyzed to FFA by pancreatic lipase resulting in AP [74]. This provides a potential explanation for why only a subset of HTG patients develops clinical HTAP. High levels of UFA are considered to play a key role in HTAP pathogenesis. It is known that the Ca^2+^ is released through the inositol 1,4,5-triphosphate receptors system and the ryanodine receptor system into the cytoplasm, thus increasing the cytosolic Ca^2+^ concentration [92]. High concentration of UFA-induced Ca^2+^ overload is not mediated by inositol 1,4,5-triphosphate [74]. However, further studies are needed to reveal the possible association between the ryanodine receptor system and UFA-stimulated Ca^2+^ overload.

### 4.4. Endoplasmic reticulum stress

#### 4.4.1. Endoplasmic reticulum stress and AP

The endoplasmic reticulum (ER) is the main site for intracellular protein processing and Ca^2+^ storage. ER is extremely sensitive to stress which is triggered by the inability to process and dispose of proteins effectively. It can result in misfolded and unfolded protein accumulation within the ER lumen and can contribute to the early stages of pancreatic injury [93,94].

#### 4.4.2. Endoplasmic reticulum stress and HTAP

**Figure 6.**
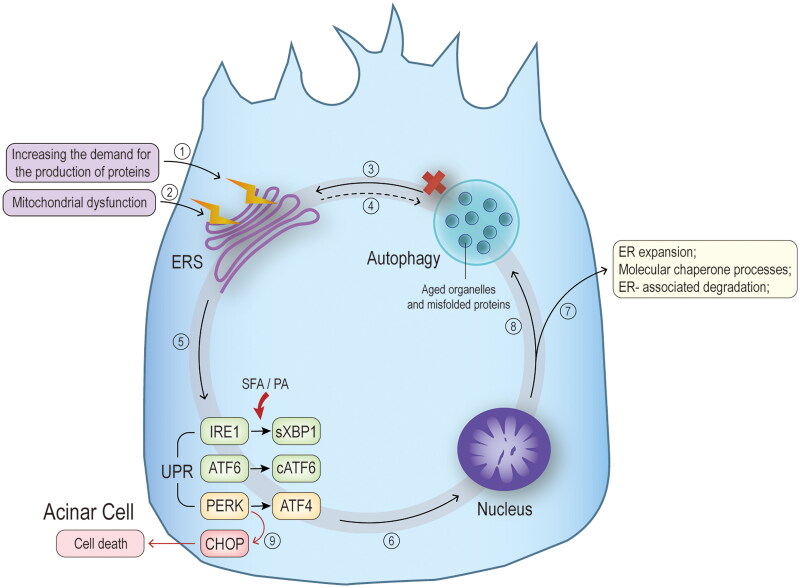
**Mechanisms of endoplasmic reticulum stress.** Extra-pancreatic pathological irritation causes endoplasmic reticulum stress (ERS) by ① Increasing the demand for production of various enzymatic proproteins on the one hand, and by reducing the ability to process and recycle unwanted proteins through ② mitochondrial dysfunction and ③ dysfunctional autophagy on the other. ④ ERS has also been found to cause impaired autophagic blood flow in animal models of hypertriglyceridaemia associated AP. ⑤ The main feature of ERS is the unfolded protein response (UPR), which includes three pathways, inositol-requiring enzyme 1 (IRE1), transcription factor (ATF) 6, and protein kinase RNA-like endoplasmic reticulum kinase (PERK), corresponding to downstream spliced X-box binding protein 1 (sXBP1), cATF6, and ATF4, respectively. Some studies have found that palmitic acid in saturated fatty acids increases the expression of UPR-related proteins and various associated cytokines by affecting the splicing of the classical IRE1-sXBP1 pathway. ⑥ The production of several transcription factors under the action of UPR will act on the nucleus to promote gene transcription for endoplasmic reticulum expansion, molecular chaperone processes required for protein folding and endoplasmic reticulum-associated degradation, ⑦ allowing the endoplasmic reticulum to meet the demands of cellular metabolism and protein synthesis. ⑧ in addition, to some extent, they also initiate and promote the process of autophagy. ⑨ nevertheless, under prolonged endoplasmic reticulum stress, the above cycle fails to restore cellular homeostasis and will induce apoptosis *via* the C/EBP homologous protein (CHOP) pathway, promoting the process of pancreatitis.

It was found that a high-fat diet per se induces ER stress, activating the inositol-requiring enzyme 1 (IRE1) a-spliced X-box binding protein 1 (sXbp1) signalling pathway (the classical pathway of EPR stress), which is closely linked to lipid metabolism and inflammation, suggesting that disorders of lipid metabolism may promote ER stress and hence, pancreatic injury [95,96]. *In vitro* studies using fatty acids to act on pancreatic exocrine cells have shown that SFA, particularly high concentrations of PA, can impact the splicing of Xbp1, increase transcriptional levels of proteins related to the UPR, elevate levels of transforming growth factor-β, and upregulate the expression of inflammatory signalling factors such as NF-κB, TNF-α, IL-6, and IL-1β [21,73,97]. Administration of the ER stress inhibitor has decreased the expression of related proteins and significantly reduced inflammatory response and injury in pancreatic acinar cells [97]. All the above studies further validate that FFAs may exacerbate the course of HTAP through the tight association of Xbp1 withER stress (Figure 6). Interestingly, SFA (e.g. PA) has a deleterious effect through exacerbating ER stress and aggravation of pancreatic stress markers in different models of exocrine pancreas cells. However, UFA (e.g. OL) has a protective anti-stress and anti-inflammatory effect [21].

ER stress is tightly associated with autophagic destruction during HTAP progression. Autophagy is completed through a series of steps that start with the enucleation of cytosolic contents within an open double membrane formed from the ER, Golgi apparatus, and plasma membrane [98]. Then, the double membrane edges meet to form an autophagosome mediated by autophagy-related proteins. Finally, the autophagosome fuses with the lysosome and the enclosed contents are degraded [88,99]. Effective autophagy eliminates misfolded, damaged proteins/organelles and inhibits inflammation [88]. Mei et al. found an elevated level of ER stress in pancreatic tissue and a significant increase in the expression of autophagic vesicles and autophagy markers in the animal models, indicating the presence of autophagic disease and impaired autophagic blood flow during HTAP progression [50]. Similar findings in the cellular models were notably significantly improved after administering ER stress inhibitors to alleviate the stress [50]. Both *in vitro* and *in vivo* studies have indicated that disruption of normal autophagy function in AP leads to abnormal activation of trypsinogen [100], ER stress, and mitochondrial dysfunction, causing necrosis of acinar cells [88].

### 4.5. Oxidative stress

#### 4.5.1. Oxidative stress and AP

**Figure 7.**
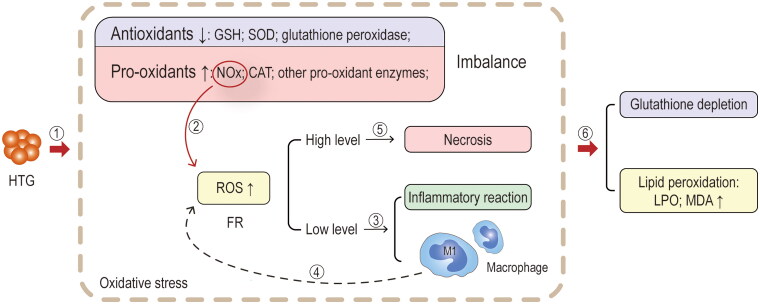
**Mechanisms of oxidative stress.** ①Animal studies have confirmed that in the hypertriglyceridaemia associated AP (HTAP) model, HTG induces the production of oxidative stress, which consists of an imbalance in the production of antioxidants (glutathione (GSH), superoxide dismutase (SOD), glutathione peroxidase) and pro-oxidants (pro-oxidant enzymes such as nicotinamide adenine dinucleotide phosphate oxidase (NOx) and catalase (CAT)) and an increased production of free radicals (FR, mainly reactive oxygen species (ROS)). ②NOx in pro-oxidants is one of the main sources of ROS. ③When ROS is mildly increased, it acts as a mediator of the inflammatory signalling pathway itself, enhancing the expression of chemokines, cytokines and adhesion factors, which in turn promote the inflammatory response; it also promotes leukocyte migration, activation and adhesion. Macrophages undergo a phenotypic shift in local infiltration, with polarisation towards the M1 type. ④Most notably, M1-type macrophages also promote cytokine production and ROS production. ⑤As the levels of ROS increase, cytotoxic effects are produced, causing pancreatic necrosis and promoting the process of HTAP. ⑥The effects of oxidative stress in the experimental model are manifested by depletion of GSH and lipid peroxidation (the metabolites are mainly lipid peroxide (LPO) and malondialdehyde (MDA)).

Oxidative stress has been identified as an important factor in the onset of AP and also contributes to the systemic inflammatory reaction, xanthine oxidase activation, glutathione, and thiol oxidation [101,102] (Figure 7). The imbalance between pro-oxidants and antioxidants leading to increased free radical formation has been defined as oxidative stress, which accelerates inflammation by recruitment and activation of inflammatory cells. The accelerated inflammatory response exacerbates oxidative stress, creating a vicious cycle [103]. Reactive oxygen species (ROS), as one of the key free radicals, have a constant and balanced production rate and scavenging capacity, maintaining redox homeostasis in cells and tissues. While low levels of reactive oxygen act as inflammatory mediators to present protective role, high levels of ROS lead to cytotoxic effects in acinar cells *via* receptor-interacting protein 3 (RIP3) pathway, resulting in positive feedback TNF-induced pancreatic necrosis [104,105]. Macrophages undergo a phenotypic shift in local infiltration, with polarization towards the M1 type [23]. Acinar cell damage and M1 macrophage polarization can lead to the development of systemic inflammatory response syndrome and multiple organ dysfunction syndrome by interacting reciprocally to form a vicious circle [98,106,107].

#### 4.5.2. Oxidative stress and HTAP

HTAP is accompanied by a decrease in the production of antioxidants (e.g. superoxide dismutase (SOD), glutathione (GSH), and glutathione peroxidase) in pancreatic tissue and an increase in the production of pro-oxidant enzymes (e.g. reduced nicotinamide adenine dinucleotide phosphate oxidase (NOx), and catalase (CAT)). The antioxidant/oxidant imbalance triggers an inflammatory response and further aggravates pancreatic injury [19,75 108,109]. According to a study by Hong et al. there is a significant increase in the levels of lipid peroxide (LPO) and malondialdehyde (MDA), which are products of oxidative stress, along with elevated serum FFA in HTAP. In addition, there was a notable reduction in the levels of SOD and GSH in pancreatic tissue, indicating the occurrence of oxidative stress and inflammatory damage during the progression of this condition [19]. Furthermore, the relationship between high-fat diet-induced hyperlipidaemia and oxidative stress and ROS is implicated as the primary factor contributing to tissue damage and the inflammatory response observed in AP [104]. Various tissues have been observed to exhibit increased expression of NOx in response to high-fat diets, leading to oxidative stress damage [110,111]. NOx is one of the main enzymes that promotes oxygen radical generation in the body, and the changes in its activity have an important impact on the level of oxygen radicals in local tissues or even the whole body [112]. Therefore, the inhibition of NOx activity reduced the degree of damage to the pancreas and kidney along with the expression of oxygen-free radicals and inflammatory factors [6]. High-fat diets may also upregulate TLR4 and exacerbate the oxidative stress of HTAP through RIP3-mediated pathways [19]. ROS is believed to be the underlying force behind necrotic and apoptotic cell death, where RIP3-mediated ROS instigates positive feedback for TNF-induced necrotizing apoptosis [105]. Furthermore, mitochondria are one of the main sources of ROS, and their components are highly susceptible to oxidative damage [113,114]. Mitochondria is the origin of damage-associated molecular patterns (DAMPs) as well, specifically mitochondrial deoxyribonucleic acid (mtDNA), which can trigger an inflammatory response once released from mitochondria into the cytoplasm [115–117]. Owing to the absence of protective histones and limited repair mechanisms, mtDNA is particularly vulnerable to oxidative stress-related damage than nuclear deoxyribonucleic acid (DNA). This impairment of mitochondrial function and integrity occurs as a result of oxidative damage to mtDNA [113,114]. Moreover, elevated levels of PA stimulation have been associated with M1 polarization of macrophages, which leads to the secretion of cathepsin S through exosomes and ultimately targets of pancreatic acinar cells, which cause inflammation and damage to pancreatic tissue [23].

### 4.6. Chemokines and cytokines

#### 4.6.1. Chemokines, cytokines, and AP

Pancreatic acinar cells are responsible for the productionof diverse chemokines that can promote the infiltration of neutrophils and subsequent monocytes into the pancreas, aggravating AP. The C-X-C motif chemokine ligand 1 (CXCL1) functions as a chemotactic factor specifically for neutrophils. And the C-X-C motif chemokine ligand 2 (CXCL2) promotes cell migration through its interaction with the C-X-C chemokine receptor (CXCR2) [118,119]. Chemotaxis-recruited neutrophils generate neutrophil extracellular traps (NETs) as a result of a specific cell death process called NETosis. NETosis entails the release of DNA networks, consisting of histones and antimicrobial proteins derived from neutrophils, such as elastase and myeloperoxidase (MPO) [79,120].NETs have been identified to contribute to several pathological processes, including ductal obstruction, activation of pro-inflammatory signals, and premature activation of trypsinogen [120,121]. The increased trypsin activity is closely associated with elevated tissue edoema, serum amylase levels, lipase levels, inflammatory cell infiltration, and acinar cell death [122]. Bae et al. demonstrated that haem oxygenase-1 can inhibit neutrophil infiltration in the inflamed pancreas by inhibiting CXCL2, highlighting the important role of chemokines in regulating inflammatory cell trafficking [123]. Monocyte chemoattractant protein 1 (MCP-1), also known as C-C motif chemokine ligand 2 (CCL2), is expressed by various cell types. Its main function is to recruit monocytes onto the sites of inflammation through tissue injury or infection [124]. After continuous chemotaxis and recruitment by upstream mechanisms, immune cells pass into the pancreas. This causes the cellular contents from necrotic and injured cells to trigger the activation of monocytes and neutrophils, resulting in inflammation propagation [98,125]. In general, the infiltration of immune cells serves as a protective mechanism and is beneficial for disease recovery. However, necrotic pancreatic cells release DAMPs comprised of various cellular contents (e.g. high-mobility group box protein 1, and self-DNA) [126,127]. DAMPs are important mediators in mediating the activation of monocytes and can also play a central role in systemic inflammation and worsen tissue damage in AP [128]. Subsequently, the short-term clearance of DAMPs is not possible, resulting in persistent activation of pattern recognition receptors on infiltrating immune cells. This leads to the production of additional inflammatory mediators, which in turn, facilitate further immune cell infiltration and amplify the overall inflammatory response [126,127]. The significance of chemokines and their receptors in the development of AP is underscored by animal model studies, demonstrating that their inhibition can prevent pancreatic and distant organ injury [129,130].

The pathways mentioned above contribute to the amplification of pro-inflammatory cytokine production, including TNF, IL-1β, IL-6, and IL-18. These cytokines can further worsen pancreatic injury and contribute to systemic inflammation [131–133]. Inflammatory cytokines (e.g. TNF and IL-6) can orchestrate the self-aggression and destruction of acinar cells [134,135]. IL-6 was found to be upregulated in a range of patients with AP and regarded as a determining factor of AP severity [136]. Likewise, the chemokine IL-6 also facilitates neutrophil infiltration and promotes anoctamin 1 (a Ca^2+^-activated Cl^−^ channel) expression *via* activating IL-6 receptor/signal transducer and activator of transcription 3 (STAT3) signal to facilitate the pathogenesis of AP [137]. Following the pro-inflammatory phase in AP, a compensatory anti-inflammatory response syndrome ensues. This phase is characterized by an abundance of anti-inflammatory cytokines such as transforming growth factor (TGF)-β, IL-4, and IL-10 [132,138]. IL-10 has the ability to decrease the production of pro-inflammatory cytokines at transcriptional level, by inhibiting the STAT3 pathway and limiting T-cell expansion. As a result, it exerts potent anti-inflammatory effects [139,140].

#### 4.6.2. Chemokines and cytokines and HTAP

**Figure 8.**
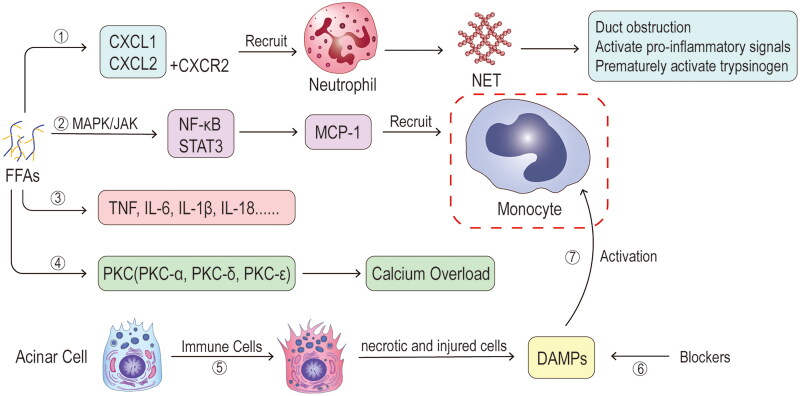
**Mechanisms of chemokines and cytokines.** ①Free fatty acids (FFAs) increase C-X-C motif chemokine ligand 1 (CXCL1) and C-X-C motif chemokine ligand 2 (CXCL2), which bind to C-X-C chemokine receptors (CXCR2) and together chemotactic neutrophil aggregation and later form neutrophil extracellular traps (NETs) that activate pro-inflammatory signals to increase the release of inflammatory factors. ②Some species of FFAs can increase monocyte chemoattractant protein 1 (MCP-1) through activation of mitogen-activated protein kinase (MAPK)/janus kinase (JAK)-mediated nuclear factor kappa B (NF-κB) and signal transducer and activator of transcription 3 (STAT3) pathways, which in turn chemotactic monocyte aggregation. ③FFAs can directly promote the release of inflammatory factors (TNF, IL-6, IL-1β, IL-18, etc.) and also indirectly increase the release of inflammatory factors through the above pathways. ④From animal models, it is concluded that FFAs are associated with an upregulation of protein kinase C (PKC) activity, which triggers calcium overload. ⑤When inflammation occurs and immune cells infiltrate the pancreas, this leads to necrosis and damage of pancreatic cells and the release of cellular contents. ⑥Damage-associated molecular patterns (DAMPs) are key cellular contents that are regulated by blockers through binding to immune cell receptors for inflammation. ⑦These cellular contents are important mediators of active monocytes, and activated monocytes play a very critical role in inflammatory injury and exacerbation.

Initially, HTAP was thought to be caused by inflammatory cascades involving chemokines and cytokines [70,141] (Figure 8). FFA is an indispensable factor for HTAP occurrence by increasing inflammatory mediators, including CXCL1, CXCL2, MCP-1, TNF-α, IL-6, and IL-10 [23,71,142]. In acute exposure to high levels of PA, primary exocrine pancreatic cells also express a pro-inflammatory state by a significant elevation in TNF-α, IL-6, TGF-β transcript, and protein levels [21]. On the other hand, Navina et al. have found that sub-lethal concentrations of LA (200 μmol/L) increase mRNA expression of TNF-α and neutrophil chemokines CXCL1 and CXCL2 [65]. Besides, OA or LA upregulate MCP-1 expression by activating the MAPK/Janus kinase (JAK)-mediated NF-κB and STAT3 pathways in glandular acinar cells [143]. MCP-1 also has the potential to serve as a distinct biomarker of HTAP, because the severity of AP can be mitigated by reducing MCP-1 expression [144]. Moreover, clinical studies have found that effective plasmapheresis improved the condition of HTAP, as it correlates with decreased levels of pro-inflammatory factors and an increase in the anti-inflammatory cytokine IL-10 [145].

The activity of protein kinase C (PKC) aligns with the degree of pancreatic injury, indicating a strong association with PKC and the exacerbation of HTAP [74,146]. Studies have shown that FFA can induce the upregulation of various PKC isoforms, e.g. PKC-α, PKC-δ, and PKC-ε. These regulate the expression of inflammatory mediators and facilitate the release of enzyme granules from acinar cells [74,146,147]. Under pathophysiological stimulation, activation of the PKC-α isoform redirects the process of exocytosis towards the basolateral membrane. PKC-δ and PKC-ε isoforms activations trigger the production of inflammatory mediators and subsequent exocytosis of enzyme granules within acinar cells *via* the NF-κB pathway [147]. The literature on the connection between PKC and HTAP remains to be explored.

### 4.7. Genetic polymorphism

For hereditary pancreatitis, heterozygous variants in two mechanistically genes of ‘trypsin-dependent pathway’, PRSS1 (encoding cationic trypsinogen) and SPINK1 (encoding pancreatic secretory trypsin inhibitor products), are said to have the effect of occurring human pathogenic variants [148,149]. Because of the rarity of highly pathogenic variants, PRSS1 and SPINK1 are not significant contributors to variation in AP susceptibility of interindividual patients with severe HTG, but may explain different HTAP risks of those ones [149]. Moreover, the increased genetic burden for HTG is considered to be associated with the risk of AP. Primary HTG is attributed to genetic defects that result in loss of function, which reduces the lipolysis of CM and further leads to triglyceride accumulation [150]. In recent years, LPL mutations have been identified in HTAP patients [93], primarily concentrated in LPL, apo C-II, apo A-V, LMF 1, and HDL-binding protein 1, resulting in LPL being non-functional or hypofunctional [151,152]. Thus, a recombinant virus with the human LPL^S447X^ variant was used in HTAP patients in early clinical treatment and temporarily reduced plasma triglyceride levels [153]. In addition, multiple studies have been performed to find the specific HTAP associated genes [154]. Chang et al. conducted a genetic analysis on a cohort of 126 HTG patients, 46 of whom had HTAP. It was discovered that the mutation rate of the cystic fibrosis transmembrane conductance regulator (CFTR) gene in HTAP patients was 26.1%. This is in contrast to a significantly lower mutation rate of 1.3% observed in patients without HTAP [155]. Later, genetic polymorphisms of the apo E subtype apo E4 and CFTR and TNF-α were also considered risk factors for HTAP [156–158].

### 4.8. Gut microbiota

There is a strong association between gut microbiota and lipid metabolism disorders as well as systemic inflammation [159]. Long-term high-fat diets cause dysbiosis of the gut microbiota, which results in increased intestinal permeability, triggering mucosal immune responses, obesity, and chronic inflammation [160]. Gut microbiota is also strongly associated with AP [161,162]. According to Huang et al. intestinal barrier dysfunction is associated with the development of AP, and HTG can potentially impact the microbiota’s ecology by influencing the expression of antimicrobial peptides (AMPs) in Paneth cells. This, in turn, can contribute to intestinal barrier dysfunction [42].

## 5. Clinical significance and future perspectives

### 5.1. Clinical significance

American College of Gastroenterology Guidelines recommend that serum triglycerides should be above 1000 mg/dL (>11.3 mmol/L) to be considered the cause of AP [8]. Based on the previous discussion on the potential mechanisms of HTAP, it can be inferred that triglycerides themselves are not inherently toxic to the pancreas. Instead, the breakdown of triglycerides by pancreatic lipase into FFAs is considered to be a critical mediator in the development of HTAP. Long-term exposure to excess SFA also shows lipotoxic effects in chronic illnesses [65]. Therefore, diagnosing and preventing HTAP at an early stage is of great clinical significance. Lipid-lowering therapy should be instituted immediately if a diagnosis of HTAP is established. Lipid-lowering management includes plasmapheresis, insulin infusion, heparin, and hemofiltration. High concentrations of UFAs may play a critical role in the underlying mechanism of HTAP [74], but OAs may act with protective effects against PA-induced lipotoxicity [66]. Thus, regulation of the lipid composition *in vivo* may provide potential prevention of the progress of this disease [163].

Emerging genetic drugs, such as alipogene tiparvovec and LCQ908, hold promise for the treatment of patients with LPL deficiency [164]. Some specific genes may be targets for diagnosis and/or therapeutic intervention based on the patient’s genetic background [154]. We also discussed that there may be some possible links between HTG, gut microbes, and AP, and a stable intestinal microbiome may bring some preventive effects to HTAP.

### 5.2. Future perspectives

Our understanding of the pathophysiology of HTAP is still in its infancy, although the association between HTG and AP has been well-established [165,166]. All the animal models reported for HTAP exhibit various degrees. Mice and rats have such high LPL activity that it is impossible to create an ideal animal model of HTG (triglyceride level > 1000 mg/dl) by simply feeding them a high-fat diet. However, genetically modified animals have solved the problem but the widespread use of animal models for HTAP is limited due to various factors, including the high cost of breeding, difficulties in reproduction, mismatches with human plasma lipids, and other related challenges. Last but not least, the lack of standardized diagnostic criteria for HTAP in animals poses a significant challenge. Different animal species may have varying criteria and treatment protocols for diagnosing and managing HTAP. This complicates research and hampers the development of consistent approaches in studying and addressing HTAP in them [39,40]. The current situation highlights the pressing need to develop a novel, stable, effective, and straightforward animal model for HTAP. Such a model would greatly facilitate the study of the underlying pathogenesis and enable the exploration of specific preventive measures. However, even promising findings of novel therapeutic agents made on animal models may not show the same efficacy in clinical trials [167]. In animal studies, the therapeutic agent is typically administered before or concurrently with the induction of AP. However, in clinical trials, treatment can only be initiated after the onset of symptoms. As a result, animal studies have primarily focused on elucidating the mechanistic pathways involved in the pathogenesis of the disease [38]. In the future, human organoids may offer enhanced and more representative models for studying AP, allowing for better translation of findings to human patients [168]. On the other hand, HTAP has a complicated pathophysiology that involves FFAs, microcirculatory disorder, Ca^2+^ overload, ER stress, oxidative stress, chemokines and cytokines, genetic polymorphism, and gut microbiota. However, limited knowledge exists regarding the specific effects of SFAs on pancreatic endocrine cells, such as beta cells, as well as the intricate interplay between the exocrine and endocrine components of the pancreas in both healthy and diseased states. Further research is necessary to comprehensively understand these interactions and their significance in the development and progression of AP and other pancreatic disorders. Additional research is required to gain a comprehensive understanding of these interactions, the implications allied with developing and progressing AP and other pancreatic disorders. This area of research is likely to be an important focus of future clinical and fundamental research [169]. Different FFAs and cellular organs in pancreatitis act at various stages of the disease process [21]. To confirm the association between the factors under investigation, it is crucial to conduct further *in vivo* and *ex vivo* studies in both animal models and human subjects. Impaired autophagic blood flow is closely related to endoplasmic reticulum stress and both factors contribute to the development of HTAP, but the pathways that synergize or feedback with each other need further study. As our knowledge of the molecular mechanisms underlying acute pancreatitis progresses, our understanding of the condition deepens and it is becoming increasingly clear that there are common key pathways allied with disease progression and development, such as DAMPs, autophagy, and monocyte polarization. However, much of the underlying genetic and molecular complexity of acute pancreatitis remains to be completely understood, and there are likely to be many more genes and pathways contributing to the HTAP that need to be elucidated. In the future, research into LPL-related gene therapy and the identification of novel HTAP-specific genes can provide insights into the underlying genetic and molecular mechanisms of the disease and pave the way for the development of novel and improved treatment approaches. As our understanding of HTAP expands, it is expected that we will be able to develop more precise and individualized approaches for diagnosing and providing optimal treatment for the condition.

## 6. Conclusions

HTAP is a complex disease with a multifactorial aetiology, and not fully understood pathophysiology. As our knowledge regarding the disease evolves, we are likely to develop more effective and targeted treatments, which can reduce the occurrence of more severe disease outcomes or prevent the disease altogether. Future research will need to focus on elucidating the underlying genetic and molecular mechanisms of the disease, as well as identifying specific pathways and targets that can be used to develop more effective treatments. This will require a multidisciplinary approach that combines genetics, molecular biology, and clinical research and will likely involve collaborations between basic and clinical scientists across multiple disciplines. Ultimately, developing more effective treatments for HTAP will require a deep understanding of the underlying pathophysiology of the disease, as well as the development of more targeted and personalized approaches to diagnosis and treatment.
